# Menopause is associated with a decrease in sexual function among women with endometriosis

**DOI:** 10.1093/sexmed/qfaf019

**Published:** 2025-04-05

**Authors:** Alexandre Vallée, Pierre-François Ceccaldi, Jean-Marc Ayoubi

**Affiliations:** Department of Epidemiology and Public Health, Foch Hospital, Suresnes 92150, France; Department of Obstetrics, Gynecology and Reproductive Medicine, Foch Hospital, Suresnes 92150, France; Department of Obstetrics, Gynecology and Reproductive Medicine, Foch Hospital, Suresnes 92150, France; Medical School, University of Versailles, Saint-Quentin-en-Yvelines (UVSQ), Versailles 92150, France

**Keywords:** menopause, FSFI, endometriosis, sexuality, pain, dyspareunia, arousal, women

## Abstract

**Background:**

Menopause-related endocrinological shifts are linked to sexual dysfunction, and women with endometriosis exhibit lower Female Sexual Function Index (FSFI) scores, indicating impaired sexual well-being.

**Aim:**

To assess the impact of menopause on sexual function in women with endometriosis.

**Methods:**

An anonymous online survey was conducted among 1586 French women diagnosed with endometriosis. The FSFI questionnaire was used to evaluate sexual function, and menopause was defined as ≥12 months of amenorrhea. Multivariable logistic regression was performed to assess the relationship between FSFI scores and menopause status, adjusting for tobacco use, education, number of symptoms, and history of surgery. Logworth analyses were used to determine the strongest components of FSFI associated with menopause.

**Outcomes:**

The primary outcome was the FSFI total score and its six domains (desire, arousal, vaginal lubrication, orgasm, satisfaction, and dyspareunia) in menopausal and non-menopausal women with endometriosis.

**Results:**

Menopausal women had significantly lower FSFI scores (15.3 vs. 16.9, *P* = 0.021). After adjustment, FSFI remained significantly lower (*P* = 0.026) in menopausal women but did not reach the established FSFI cutoff for sexual dysfunction (*P* = 0.451). Stratified analysis by age showed a steep decline in FSFI between 46 and 50 years, partial improvement at 51-55 years, and further decline after 55 years, particularly in arousal, orgasm, dyspareunia, and satisfaction. Arousal (logworth = 4.53, *P* < 0.001) was the most affected domain, followed by satisfaction (logworth = 1.81, *P* = 0.015).

**Clinical Implications:**

Arousal appears to be the key determinant of sexual function decline in menopausal women with endometriosis, highlighting the need for targeted interventions such as hormone therapy, pain management, and sexual counseling.

**Strengths & Limitations:**

The study benefits from a large sample size and validated FSFI assessment but is limited by selection bias from online recruitment, self-reported diagnosis of endometriosis, and lack of hormonal status confirmation. The cross-sectional design prevents causal inferences.

**Conclusion:**

Menopause is associated with a decline in FSFI scores among women with endometriosis, with arousal being the most affected domain, underscoring the need for further research on personalized management strategies for sexual dysfunction in this population.

## Introduction

The prevalence of sexual dysfunction in postmenopausal women remains uncertain due to ongoing debates over its definition and diagnostic criteria, with reported rates ranging from 35.9% to 86.5% across various countries.^1^ Factors contribute to diminished sexual function, including psychological and emotional well-being, aging, persistent health conditions, metabolic syndrome, and the menopausal phase itself.^2^ Climacteric symptoms, prompted by endocrinological shifts during menopause, are linked to sexual dysfunction.

Sexual dysfunction is a well-documented but often under recognized consequence of endometriosis, affecting multiple domains of female sexual health, including desire, arousal, orgasm, and pain during intercourse (dyspareunia).^3^ Women with endometriosis report significantly lower Female Sexual Function Index (FSFI) scores compared to those without the condition, highlighting the profound impact of chronic pelvic pain, inflammation, and hormonal dysregulation on sexual well-being.^4^ Dyspareunia, a hallmark symptom of endometriosis, is often caused by deep infiltrating lesions, adhesions, and nerve involvement, making sexual activity uncomfortable or even intolerable. Additionally, psychological distress, including anxiety, depression, and fear of pain, further contributes to sexual dysfunction, leading to avoidance behaviors and reduced sexual satisfaction.^5^ Given that menopause introduces additional challenges, such as declining estrogen levels, vaginal atrophy, and neurovascular changes, it is crucial to explore how these factors interact in women with endometriosis. By examining the specific effects of menopause on sexual function in this population, this study aims to provide a more comprehensive understanding of the compounded impact of endometriosis and hormonal changes on female sexuality. While data on perimenopausal women are scarce, there is an indication that sexual dysfunction becomes more prevalent during this stage of life. Risk factors for sexual dysfunction such as climacteric symptoms are known to increase in this period. Women diagnosed with endometriosis exhibited significantly lower FSFI scores in comparison to their healthy counterparts, indicating impaired sexual well-being.^4^ Thus, the goal of the present study was to assess the impact of menopause on sexual functioning among women with endometriosis.

## Methods

### Study design and participants

An anonymous online survey was developed by our team.

The study link was disseminated via social media where participants were asked to forward this link to others they know. The anonymity of participants was ensured. All registrants were free to accept or decline the invitation, with no monetary reward received in return. Participants were also informed that they could withdraw at any time. A research protocol was conducted to obtain approval from an ethical committee. The distribution of the questionnaire was occurred between November 2023 and January 2024 in France on social media (Instagram, Facebook, in French language). The study was approved by the Foch IRB: IRB00012437 (approval number: 23-07-05) on 18 July 2023. Informed consent was obtained from all participants.

Questionnaire and measuring instruments.

The questionnaire was divided into the following sections:

- Sociodemographic questions [marital status, age, educational level, children, body mass index (BMI) level calculated as weight (in kg) divided by height squared (in meters) and categorized as high (BMI > 30 kg/m^2^), moderate (BMI between 25 and 30 kg/m^2^), and low (˂25 kg/m^2^)].
- Questions related to the disease (diagnosis, symptoms, treatment, age of diagnosis etc.)
- Symptoms of endometriosis were defined as: pain during sexual intercourse, Abnormal or heavy menstruation, Infertility, pain during urination during periods, Pain during bowel movements during periods, Other digestive issues (diarrhea, constipation, nausea), worsening pain over time, pain, particularly excessive menstrual cramps that are felt, and Others.
- Menopause was defined as no periods from ˃12 months.
- FSFI questionnaire: The FSFI contains 19 items and collects data on 6 domains of sexual function: desire, arousal, vaginal lubrication, orgasm, satisfaction, and pain. For each domain except the pain domain, the item scores range from 0 to 5. Higher item scores indicate better function. Items in the pain domain are coded by a descending scale. To obtain the total FSFI score, the item scores within each domain are added and then multiplied by a correction factor. The resulting scores within each of the six domains are added to obtain a total FSFI score. Higher scores reflect better sexual function.^6^ Cut-off of FSFI was defined as 26.55.^2^

### Statistical analysis

Characteristics of the study population were described as the mean standard deviation (SD) for continuous variables. Categorical variables were described as numbers and proportions. Comparisons between groups were performed using the Mann–Whitney test or t Student test for continuous variables. Pearson’s *χ*^2^ test was performed for categorical variables. Associations between FSFI level and cutoff of FSFI and menopause status were adjusted for covariables with *P*-value <0.20 in univariable analysis. Logworth analyses were performed to assess the order of the strength relationship between all the components of FSFI with menopause. Statistics were performed using SAS software (version 9.4; SAS Institute, Carry, NC). A *P-*value <0.05 was con- sidered statistically significant.

## Results

**Figure 1:**
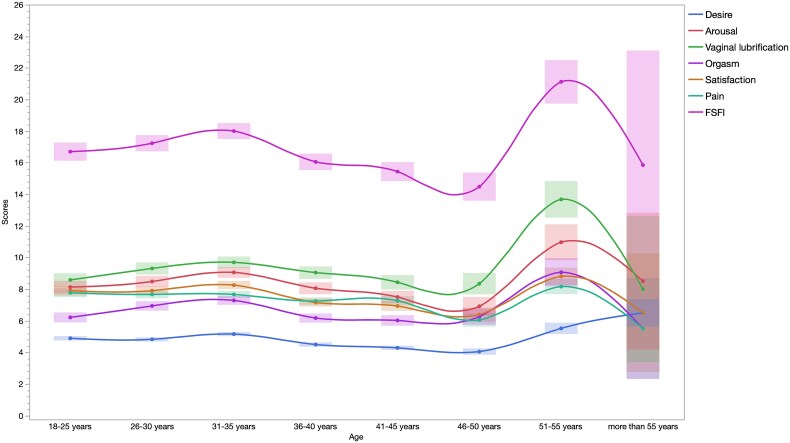
Range scores of FSFI and each component according to age range. FSFI, Female Sexual Function Index.

Women were divided into two groups (menopaused, N = 206 and non-menopaused, N = 1378). We observed no difference between these two groups for couple status, having children, pain during intercourse, BMI level and for the cutoff of FSFI. Menopaused women were mainly smoking users, showing higher endometriosis symptoms, with less educational level and lower FSFI levels (15.3 vs. 16.9, *P* = 0.021, respectively) (Table 1). None of the women included in the study were undergoing hormone replacement therapy. After adjustment for tobacco, surgery, number of endometriosis symptoms, and education level, FSFI level remained significantly lower in menopaused women (*P* = 0.026) but not cutoff of FSFI (*P* = 0.451) (Table 2). When considering the stratification of age, we observed no significant difference in FSFI level (*P* = 0.476) and in cutoff of FSFI (*P* = 0.235). We showed a steep decline in women 46-50 years of age, partially increased in the 51 to 55-age group, and subsequently declined in women aged ˃55 years for arousal (6.9, 10.9, and 8.5, respectively) for orgasm (6.3, 9.1, and 5.5, respectively), for dyspareunia (6.0, 8.2, and 5.4, restively), satisfaction (6.4, 8.8, and 6.5, respectively), and FSFI levels (14.5, 21.1, and 15.9, respectively) (Figure 1). Arousal (logworth = 4.53, *P* < 0.001) was the main factor component of FSFI associated with menopause compared to satisfaction (logworth = 1.81, *P* = 0.015), orgasm (*P* = 0.202), desire (*P* = 0.241), vaginal lubrification (*P* = 0.303), and dyspareunia (*P* = 0.639) (Table 3).

**Table 1:** Characteristics of the study population.

	**Non-menopaused women N = 1378**		**Menopaused women N = 206**		
	**N**	**%/SD**	**N**	**%/SD**	***P*-value**
Couple	1089	79.03%	156	75.73%	**0.282**
Having children	653	47.59%	106	51.46%	0.301
Tobacco smoking	330	24.05%	80	39.22%	<0.001
Treatment against endometriosis	575	41.79%	104	50.98%	0.013
Surgery for endometriosis	189	13.72%	50	24.27%	<0.001
Number of endometriosis symptoms	5.7	1.8	5.1	2.1	<0.001
Pain during intercourse	1084	78.66%	170	82.52%	0.203
Education					<0.001
Moderate	349	25.33%	38	18.63%	
High	275	19.96%	24	11.76%	
Low	754	54.72%	142	69.61%	
BMI level					0.328
High	308	22.48%	56	27.18%	
Moderate	353	25.77%	50	24.27%	
Low	709	51.75%	100	48.54%	
Cutoff FSFI<26.55	1178	85.49%	178	86.41%	0.725
FSFI	16.9	9.22	15.3	8.83	0.021
Desire	4.78	2.27	4.37	2.17	0.016
Arousal	8.52	6.4	6.52	5.68	<0.001
Vaginal lubrification	9.26	6.92	8.14	6.59	0.029
Orgasm	6.74	5.19	5.69	4.75	0.006
Satisfaction	7.61	4.71	7.45	4.75	0.646
Dyspareunia	7.4	4.25	7.6	4.47	0.532

Abbreviations: BMI, body mass index; FSFI, Female Sexual Function Index.

**Table 2:** Multivariable regression analysis for menopause status.

**Parameters**	**OR 95%CI**	***P*-value**
FSFI per unit	0.98 [0.97– 0.99]	0.027
Symptoms per unit	0.84 [0.77– 0.91]	<0.001
Surgery	1.99 [1.38– 2.89]	<0.001
Education		
High	0.63 [0.43– 0.93]	0.020
Moderate	0.51 [0.32– 0.82]	0.005
Low	Ref.	
Tobacco smoking	2.05 [1.48– 2.81]	<0.001
FSFI cutoff<26.55^a^	0.84 [0.53– 1.31]	0.451

a In a separate regression model.

Abbreviation: FSFI, Female Sexual Function Index.

**Table 3:** Logworth analysis for components of FSFI according to menopause status.

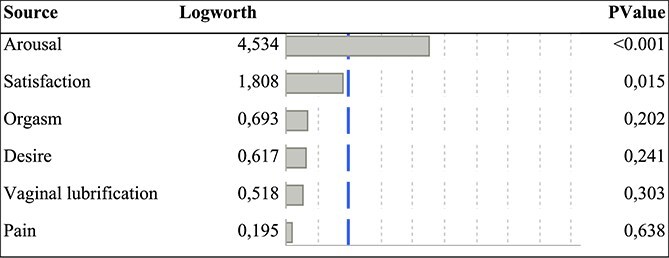

Abbreviation: FSFI, Female Sexual Function Index.

## Discussion

Our data indicated a reduction in the FSFI scores with menopause status, aligning with previous reports that this transitional period adversely affects sexual function, likely due to the reduction in central and peripheral sex hormones.^7-10^ As observed in our study (range = 86.4% in menopause), sexual dysfunction was ranging from 35.9% to 86.5% across countries.^1^^,^^2^^,^^11-13^ Our findings also revealed a distinct association between menopausal status and specific sexual function domains like arousal, which appears to be affected by hormonal levels. Supporting this notion, individuals using systemic hormone therapy exhibited higher arousal scores in the FSFI compared to those applying local vaginal treatments, suggesting that the impact of local treatments might be through changes in local vaginal conditions. Alterations in hormone levels, including the reduction of estrogen and androgens, can impact sexual organs and various bodily systems, thereby affecting sexual functionality. There is a widely acknowledged understanding among researchers regarding the impact of diminished estrogen levels on the decline in sexual function.^12^ Furthermore, the influence of androgens on sexual health has been explored, revealing that lower testosterone levels can be indicative of sexual dysfunction, particularly in naturally menopausal women.^14^ Another study highlighted that during the initial stages of menopausal transition, the levels of free plasma testosterone were identified as the key determinant of the overall sexual function score. Contrastingly, in the early phase of postmenopause, which encompasses the first 5 years after the last menstrual cycle, levels of dehydroepiandrosterone sulfate (DHEAS) and estradiol were found to be significant indicators of overall sexual function.^15^

The literature presents varying views on whether age acts as an independent factor in female sexual function.^16-18^ Factors such as personal relationships, economic conditions, cultural influences, spiritual convictions, overall well-being, and the availability of a sexual partner play significant roles in shaping women's perceptions of their sexuality during menopause.^19^

Regrettably, insufficient knowledge often leads women to overlook the connection between hormonal fluctuations and alterations in sexual function. A number of women hold the belief that their sexual functionality remains unchanged for an extended period post-menopause, attributing any sexual dissatisfaction entirely to their partners.^20^ While personal backgrounds and relational dynamics can account for certain variations observed during menopause, they do not explain all the changes encountered.^21^

In our analysis, age did not show a direct correlation with FSFI scores. Nonetheless, a significant drop in FSFI scores, including aspects like arousal, orgasm, dyspareunia, and satisfaction, was observed in women aged 46-50. The temporary improvement in FSFI scores in women aged 46-59 might be linked to the hyperestrogenism commonly associated with perimenopausal ovarian dysfunction.^2^

The observed reduction in FSFI scores, particularly in the arousal domain among menopausal women with endometriosis, is likely influenced by a complex interplay of hormonal decline, chronic pain, and psychological distress.^22^ While the study primarily attributes the loss of arousal to low ovarian steroid levels during menopause, it is important to recognize that endometriosis itself contributes to sexual dysfunction through persistent pelvic pain, dyspareunia, and tissue scarring**.** Chronic pain, independent of hormonal status, can lead to sexual avoidance, increased anxiety, and neuromuscular dysfunction, all of which may suppress arousal and sexual response.^23^ Additionally, menopause introduces genitourinary changes, including vaginal atrophy and reduced lubrication, which further exacerbate discomfort during intercourse. The combined burden of endometriosis-related pain and menopause-associated hormonal decline may result in a greater-than-expected deterioration in sexual function.^3^^,^^24^ Future research should explore the relative contributions of pain, hormonal factors, and psychological distress to sexual dysfunction in this population, as well as potential interventions, such as pain management strategies, localized estrogen therapy, and pelvic floor rehabilitation, to improve sexual well-being in postmenopausal women with endometriosis.

Prior research has indicated that menopausal symptom severity can adversely affect sexual activity and gratification.^2^^,^^25-28^ A longitudinal assessment of the FSFI score in both premenopausal and postmenopausal women revealed that the initial levels in the desire and arousal domains of the FSFI were key predictors of sexual function changes, as observed in our study. In our study, we found that arousal was the main component determining sexuality function in this period. Previous studies have shown that arousal was mainly associated with androgen levels.^29^ Despite some reports of the beneficial effects of androgen therapy on sexual dysfunction and other symptoms of androgen-deficiency syndrome, as well as its proof of being healthy in short-term uses (6 months), its long-term effects are as yet unknown.^7^ Elements associated with these aspects could be crucial in shaping female sexual function. Previous findings indicated a negative correlation between arousal and the incidence of vaginal dryness.^2^ This evidence, along with previous observations, underscores the significant influence of vaginal dryness on the entirety of female sexual health. It further implies that interventions aimed at preventing vaginal dryness could prove advantageous not only for premenopausal but for menopausal women as well.

### Limitations

As the link of the questionnaire was promote by social media, we are unable to provide a response rate and data collection without information of localization of respondents. These factors may impact the generalizability of the observed results. Our study was internet-based, which introduces the possibility of selectivity bias. Being a cross-sectional study, it does not allow for the establishment of causality. The questionnaire was designed to be simple and easy to answer, limiting our ability to evaluate daily habits.

The diagnosis of endometriosis in this study was based on self-reported data collected through an anonymous online survey. No confirmatory medical examinations, such as laparoscopic surgery or imaging studies, were required to verify the diagnosis. This introduces the possibility that only women with more severe symptoms or those who had previously received a formal medical diagnosis participated in the study, potentially leading to a selection bias. This could result in an overestimation of the impact of menopause on sexual dysfunction, as these women may already experience significant sexual impairment due to chronic pain, dyspareunia, and other symptoms associated with severe endometriosis. Moreover, the absence of a validated measure for endometriosis severity introduces potential biases in the study results. Without an objective classification, the findings may primarily reflect the experiences of women with more symptomatic or severe cases, which could lead to an overestimation of the impact of menopause on sexual function. The study was conducted exclusively among French-speaking women, which introduces a potential cultural and linguistic bias. Sexual function, perceptions of menopause, and attitudes toward endometriosis can vary significantly across different cultural, social, and healthcare contexts. The study exclusively recruited participants via Instagram and Facebook, which introduces a potential selection bias. Women who do not use social media, or who do not follow relevant groups related to endometriosis or menopause, would have been unaware of the survey and thus excluded from participation.

## Conclusion

In our study we found that FSFI was decreased in menopaused women, and that arousal was a main player in determining female sexuality in this period. Prospective studies should be investigated to better understand this association and whether selective treatment of menopause could impact this relationship.
